# Influence of Citrus Fruit Waste Filler on the Physical Properties of Silicone-Based Composites

**DOI:** 10.3390/ma16196569

**Published:** 2023-10-06

**Authors:** Maciej Mrówka, Dawid Franke, Martin Ošlejšek, Mariola Jureczko

**Affiliations:** 1 Department of Material Technologies, Faculty of Materials Engineering, Silesian University of Technology, Krasińskiego 8, 40-019 Katowice, Poland; 2 Material Innovations Laboratory, Silesian University of Technology, Krasińskiego 8, 40-019 Katowice, Poland; 3 Department of Geoengineering and Resource Exploitation, Faculty of Mining, Safety Engineering and Industrial Automation, Akademicka 2A, 44-100 Gliwice, Poland; dawid.franke@polsl.pl; 4 Department of English and American Studies, Faculty of Arts, Palacký University Olomouc, Křížkovského 10, 779 00 Olomouc, Czech Republic; oslema00@upol.cz; 5 Department of Theoretical and Applied Mechanics, Faculty of Mechanical Engineering, Silesian University of Technology, Konarskiego 18A, 44-100 Gliwice, Poland; mariola.jureczko@polsl.pl

**Keywords:** silicone, composite, casting, organic fillers, citrus, mechanical properties, abrasion

## Abstract

Silicones have been used as protective coatings due to their resistance to hydrolytic degradation and UV (ultraviolet) degradation. There is a growing problem with managing organic waste, which can be used as fillers in composites. This research demonstrated the use of organic waste from citrus peels, including grapefruit, lime, lemon, and orange peels. Silicone-based composites were prepared by gravity-casting using 2.5, 5, and 10 wt.% waste filler. Samples made from the composite panels were subjected to static tensile, density, hardness, pin-on-disc, and Schopper–Schlobach abrasion tests. The test results showed that lower tensile strength values characterized the composite materials compared to the silicone used as a filler. All materials had greater hardness than the silicone without the addition. At the same time, composites with a mass density of the filler of 2.5 and 5 wt.% showed more excellent abrasion resistance than the silicone used as a matrix. This research showed that the samples containing 2.5 wt.% grapefruit filler had the best mechanical properties and the lowest abrasive wear.

## 1. Introduction

In recent years, composite materials have gained popularity due to their many advantages, such as their low density and the ease and economy of their production [1,2,3]. Polymer-based composite materials are defined as materials in which the matrix is a polymer material, and the filler is made of organic or inorganic particles that are immiscible with the polymer [4,5,6,7]. Researchers around the world have taken steps to improve the operational properties of this group of materials by using various types of reinforcements, e.g., fibers [8,9,10,11]. A reinforcement forms a composite that is more attractive than pure plastic due to its low density, greater strength, stiffness, and increased abrasion resistance [12,13,14,15,16]. The newly developed materials and their possible degradation products must also be non-toxic to normal cells in the human body [17,18,19].

However, polymer composites reinforced with synthetic fibers also have considerable disadvantages. For example, they are unfriendly to the environment, as they are non-biodegradable, and some of them (e.g., glass, aramid, and ceramic fibers) pose oncogenic risks during manufacturing processing [20,21,22]. A practical environmental protection solution may be replacing synthetic fillers with natural ones [21,22,23,24]. Composites reinforced with fibers of organic origins also offer good strength, lightness, composite stiffness, low costs, a low friction coefficient, proper abrasive and erosive wear resistance, and, most importantly, biodegradability (including lower disposal costs) [25,26]; therefore, scientists are conducting more research into biocomposites. This is in line with increasing environmental awareness and the concepts of sustainable development (responsible waste management and designing products for recycling) [27,28,29]. Furthermore, this research complies with the circular economy strategy promoted by the European Commission and stipulates waste prevention and reuse [29].

Various types of waste can produce fillers for biocomposites, such as industrial, construction, and biological waste, to which several research works have already been devoted [21]. An interesting idea is using biological materials, e.g., parts of plants [22]. Examples of such applications include walnut shells, hazelnut shells, and sunflower seed shells to improve the stiffness of epoxy resins [29]. Waste from processing oranges has been used as a filler to enhance mechanical properties [24] and resistance to sliding wear [30]. Pineapple leaves [31] and peanut shells have been used in insulation [32].

Using silicones as the matrix for composites is a relatively new concept. In the literature, there are few examples of their use as the matrix to produce composites with various properties. In the last five years, however, scientific articles have presented silicon-based composites in many fields. The literature describes composites based on silicones in which ground quartz and wollastonite were used as fillers and cable sheaths [33]. In a study from 2013, the conductivity and mechanical properties of the resulting composites were improved by adding nickel to silicone [34]. Masłowski et al. in 2015 showed that adding magnetite and ionic pleats improved the mechanical properties of composites in which silicone played the role of the matrix [35]. Further research by Masłowski et al. in 2016 showed that adding iron oxide Fe_3_O_4_ improved the mechanical properties of silicone-based composites [36]. Imiela et al. showed that adding ceramic powders to silicones did not reduce the physicochemical properties of the resulting composites [37]. In 2019, Jin et al. used graphene oxide as a filler for silicone composites. Adding 0.36 wt.% graphene oxide protected the silicone composite against fouling by marine plants [38]. In a 2019 study, phosphorus–silicone composites were used in white light-emitting diodes (LEDs) [39]. Beter et al. investigated the effect of fibers on the mechanical behavior of fiber-reinforced silicones under cyclic loading [40]. Song et al. studied ceramic composites of silicone rubber with added ZrSi_2_ and used them as protective coatings [41]. The Silesian University of Technology research using silicones as the composite matrices resulted in several results in which composites with organic and non-organic filling were tested. The study used inorganic zinc and manganese wastes from the steel industry as fillers in silicone-based composites [42,43].

This paper aims to test the effect of citrus residues (grapefruits, key lime, lemon, and orange peels) as fillers for silicone-based composites. This study aims to determine how a given waste and its content affect the operational properties of the material (static tensile test, density, hardness, and resistance to abrasive wear).

## 2. Materials and Methods

### 2.1. Materials

**Figure A1:**
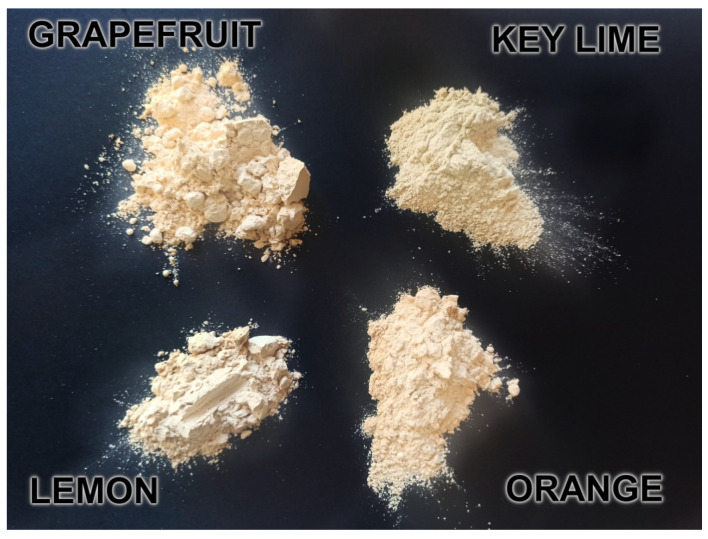
Materials used as fillers.

Mold Star™ 30 silicone produced by Smooth-On, Inc. (Macungie, PA, USA) was used as the matrix. The properties of Mold Star™ 30 silicone are shown in Table A1. The silicone fillers used in this research were waste citrus peels, which were generated in households and collected by the authors of this publication. The peels of the grapefruit (*Citrus paradisi*), key lime (*Citrus aurantifolia*), lemon (*Citrus limon*), and orange (*Citrus sinensis*) were used. The materials thus prepared were used as fillers (Figure A1). The distilled water used for density analysis was produced using an HLP-10 demineralizer (Hydrolab, Straszyn, Poland).

### 2.2. Methods

#### 2.2.1. Fillers Characterization

The grapefruit, key lime, lemon, and orange peels were first dried in a KC-65 laboratory drier (Premed, Warsaw, Poland) at 85 °C for 24 h. Subsequently, dried peels were ground using a vibratory disc mill (Testchem, Pszów/Radlin, Poland). The following process parameters for each type of citrus peel were used: a mill load of 10 g/min and a grinding time of 1 min. The particle size analysis of the ground products was carried out using screens with mesh sizes 0.32 mm, 0.22 mm, 0.18 mm, 0.16 mm, 0.125 mm, 0.065 mm, and 0.03 mm (Fritsch, Idar-Oberstein, Germany). Tap densities of the fillers were assessed by the method described in ISO 3953. The measurements were carried out using cylinders of 100 cm^3^ capacity. The tapping frequency was ca. 150 taps/min. The time of each measurement was 7 min. Bulk density was determined after the sample was inserted into the cylinder.

#### 2.2.2. Composite Preparation

The composites were prepared by gravity casting with filler contents of 2.5, 5, and 10 wt.%. The composites are marked as shown in Table 1. Before introducing fillers into the silicone matrix, it was gradually heat-treated at 80 °C for 180 min until a constant weight was obtained. The silicone components were mixed with the fillers on a high-shear mixer with a mixing speed of 500 rpm. Seventy-two hours after being poured into molds, samples were cut by punching, and the obtained samples were subjected to mechanical and physical tests. All tests were conducted at room temperature (22 °C) and with a humidity of 50%. The surface characterization of composite materials was carried out using a Phenom ProX scanning electron microscope (SEM) at an accelerating voltage of 10 kV.

#### 2.2.3. Tensile Test

Tensile tests were conducted according to EN ISO 527-1 for five samples (type 5-B) cut from the composites and silicone samples [44]. The tests were conducted on an Instron 4465 (Instron, Norwood, MA, USA) tensile tester equipped with a mechanical contact extensometer. The test speed was 500 mm/min. The tensile strength and elongation at break were determined.

#### 2.2.4. Density Test

The densities of composites were measured on a scale by EN ISO 1183-1:2006 using five samples from each material [45]. The density was determined by the hydrostatic weighing of composite polymer materials, which involved weighing the test sample with an Adventure Pro AV264CM (OHAUS Europe GmbH, Nänikon, Switzerland) analytical balance with a density measurement kit. Each sample was weighed twice. The first measurement was carried out with the sample placed on the pan and surrounded by air. The second measurement was carried out for a sample immersed in a liquid of a known density. During the tests, water was used as the liquid with a known density *d* = 0.997 g/cm^3^.

The density was determined using Formula (1):

(1) $$d=d_{W}\frac{m_{1}}{\left(m_{1}-m_{2}\right)}$$

where:

*d_W_*—density of water [g/cm^3^];

*m*_1_—dry sample mass [g];

*m*_2_—wet sample mass [g].

#### 2.2.5. Hardness Test

Shore A hardness tests were carried out by EN ISO 7619-1:2010 [46]. The measurements were made with a hardness durometer Shore A type Zorn (Zorn Instruments GmbH and Co., Hansestadt, Germany). Five measurements were made on each sample.

#### 2.2.6. Pin-on-Disc

Abrasion tests were performed using a CSM Tribometer (Needham, MA, USA) by ASTM G99 [47]. The surfaces of the tested samples and counter samples were cleaned with isopropanol. A tungsten carbide (WC) ball with a diameter of φ = 6 mm was used as a counter sample. The values of the normal load F_n_ equal to 0.5 N were used. The test was carried out with a linear velocity of 5 cm/s and a total distance of 30 m. The measurement was carried out three times for each tested material. The frictional force (F_n_) was measured for all tested materials during the test.

#### 2.2.7. Abrasion Resistance Tests

Abrasion resistance tests were carried out on an APG Schopper–Schlobach apparatus (APG Germany GmbH, Friedberg, Germany) in accordance with the EN ISO 4649:2007 standard [48]. Sandpaper (80 grit) was used, which was wound on a roller with a diameter of 150 mm and rotated at 40 rpm. Abrasion resistance was determined for three samples. Abrasion resistance (abrasive wear), i.e., the volume loss relative to a standard sample, was determined based on Formula (2):

(2) $$\Delta V=\frac{m_{1}-m_{2}}{d}\;$$

where:

*m*_1_—mass of sample before abrasion [g];

*m*_2_—sample mass after abrasion [g];

*d*—sample density [g/cm^3^].

## 3. Results and Discussion

### 3.1. Fillers Characterization

The yield of grain classes for each type of citrus peel is provided in Table 2. The negligible amount of grains with dimensions larger than 0.22 mm were removed to prevent the formation of agglomerates and huge grain concentrations.

The fillers had similar bulk densities of approximately 0.45 g/cm^3^ (Table 3). The lowest tap densities were for grapefruit and orange powder, 0.69 g/cm^3^ and 0.78 g/cm^3,^ respectively. The key lime and the lemon powder had nearly the same tap density, 0.84 g/cm^3^. The cause of variations in tap density could be the differences in albedo peel sizes.

### 3.2. SEM Analysis

**Figure 1:**
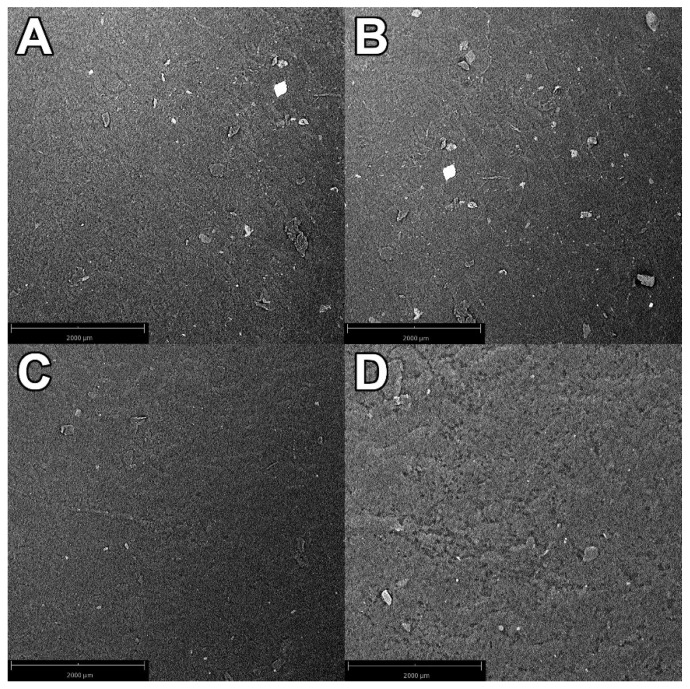
Scanning electron microscope (SEM) images of 10G (**A**), 10K (**B**), 10L (**C**), and 10O (**D**) composite materials.

The SEM images of 10G, 10K, 10L, and 10O composite materials are shown in Figure 1.

SEM images of the surface of the obtained composite materials with the content of fillers equal to 10% by mass were taken. The surface of all composite materials is smooth; there are no matrix cracks or empty spaces at the contact point of the filler grains with the matrix. The grains of the composite material on the surface are there due to falling in the gravity casting process. This proves that the process of obtaining the composite material was carried out correctly because the obtained material does not show any discontinuity, and the polymeric material combines with the filler grains. In addition, no agglomerates of filler particles can be seen on the material’s surface, which may indicate that the fillers were evenly distributed in the composite matrix. The largest grains of the filler, which can be seen in the pictures, have a diameter of about 300 µm, which corresponds to the size of the filler grains contained in Table 2. It can be presumed that the white spaces on the border between the filler and the matrix are carbon from the organic fillers, which precipitated on the silicon from which the silicone used as the polymer matrix is built.

### 3.3. Tensile Tests

**Figure 2:**
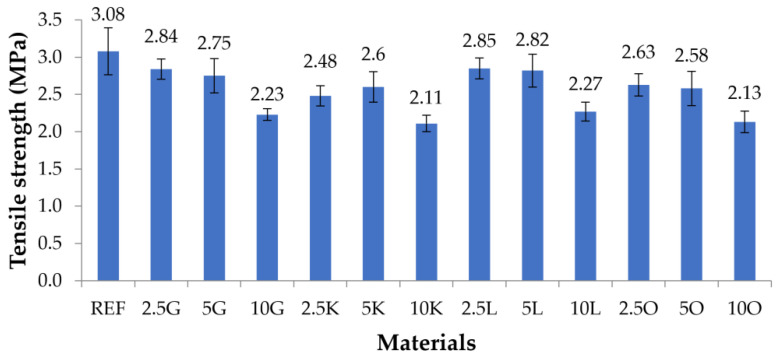
Tensile strengths of the tested materials.

**Figure 3:**
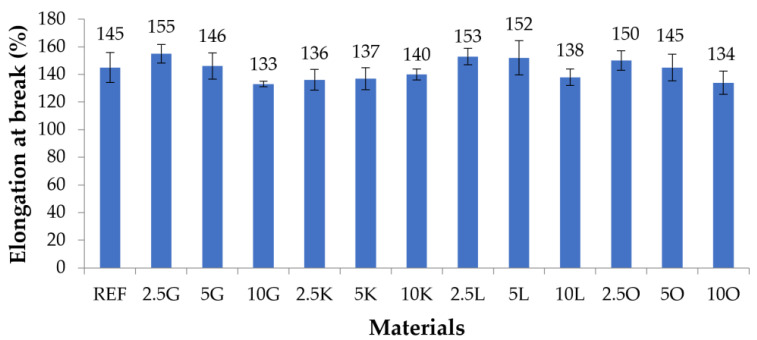
Elongation at break of the tested materials.

The results of mechanical tests for composite materials and pure silicone (REF) are presented in Figure 2 and Table 4 (tensile strength) and Figure 3 and Table 5 (elongation at break).

The highest tensile strength (3.08 MPa) was found for samples from the material without the REF filler. The tensile strengths of the remaining composite materials were lower than the native samples. Converging trends were observed for the composite materials in which grapefruit and lemon peels were the filler. The tensile strength values decreased upon increasing the filler content in the composite (for samples with grapefruit: 2.5G: 2.84 MPa, 5G: 2.75 MPa, and 10G: 2.23 MPa). In the case of samples with orange peels, the trend was similar: 2.5O: 2.63 MPa, 5O: 2.58 MPa, and 10O: 2.13 MPa. For samples containing lime in the matrix, the highest tensile strength was found for samples with 5 wt.% filler content (2.6 MPa), and a slightly lower value was recorded for samples with 2.5% lime (2.48 MPa). The lowest value was measured for samples with 10% lime (2.11 MPa). In the case of samples with lemon, the samples with 2.5 and 5 wt.% filler had similar tensile strength values of 2.85 and 2.82 MPa, respectively. For samples containing 10 wt.% lemon, the tensile strength was 2.27%. For all tested materials, the lowest tensile strengths were recorded for the samples with the highest filler content (10 wt.%). The lowest values for 10K (2.11 MPa) and 10O (2.13 MPa) were about 30% lower than the highest tensile strength measured for the REF sample (3.08 MPa).

The samples without filler showed an elongation at a break of 145%. For samples with grapefruit filler, an increase in the elongation at break was observed for samples with 2.5 wt.% filler content (155%). The elongation at break for the samples with 5 wt.% grapefruit was 146%, close to the value recorded for the REF sample. The lowest value was recorded for samples with 10% grapefruit content (133%). For the grapefruit samples, the elongation at break decreased upon increasing the filler addition. A similar trend was also noticed for the samples with added oranges. For samples with 2.5 wt.% filler, the value was 150%; for samples with 5 wt.%, it was 145%; and for samples with 10 wt.%, it was 134%. For the samples with lime, the elongation at break values were similar in the 2.5K (136%) and 5K (137%) samples. For samples containing 10% lime in the matrix, the highest value was recorded in the group with the lime filler (140%). For composite materials in which lemon acted as the filler, the elongation at break values for samples containing 2.5 and 5% filler were also like each other, with 153 and 152% values. The value for the sample containing 10 wt.% lemon was 138%. The highest elongation at break was recorded for samples with 2.5 wt.% grapefruit (155%), approximately 7% greater than the REF sample. The lowest value was recorded for samples with 10 wt.% grapefruit and oranges (133 and 134%). These values were approximately 8% lower than the samples without added fillers.

Similar trends were observed for the tensile strength and elongation at the break of the examined materials. For both grapefruit and lemon samples, both the tensile strength and elongation at break were the highest for the samples with the lowest filler content (2.5 wt.%). They decreased upon increasing the filler content in the samples. For samples filled with lemon, the 2.5 and 5 wt.% materials also had similar tensile strengths and elongation at break values. For samples with a lemon content of 10 wt.%, the filler content caused the tensile strength and elongation at break values to drop significantly below those of the samples with 2.5 and 5 wt.% lemon filler.

### 3.4. Density Test

**Figure 4:**
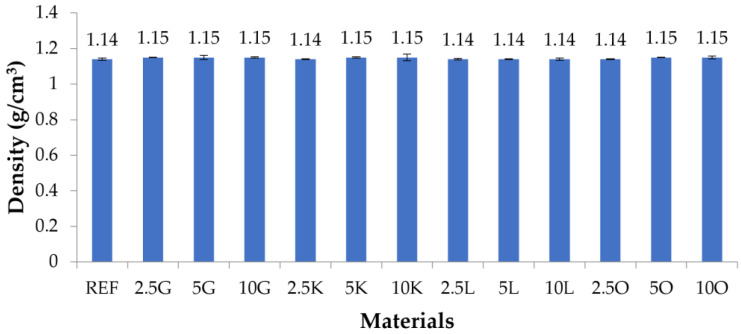
The densities of the tested materials are determined by the hydrostatic weighing method.

The density values are presented in Figure 4 and Table 6. The density of the sample without filler was 1.14 g/cm^3^. The density of grapefruit filler samples was 1.15 g/cm^3^ for all filler contents. The same was valid for lemon. Regardless of the filler content in the model, the density was 1.14 g/cm^3^. For models with lime and orange fillers, the density of the samples containing 2.5 wt.% filler was 1.14 g/cm^3^, the same as the REF material. For both groups of composite materials, the samples with 5 and 10 wt.% filler contents had a density of 1.15 g/cm^3^. These values were 1.14 and 1.15 g/cm^3^, which are very similar (a difference of approx. 0.9%) and should be treated as the same.

### 3.5. Hardness Test

**Figure 5:**
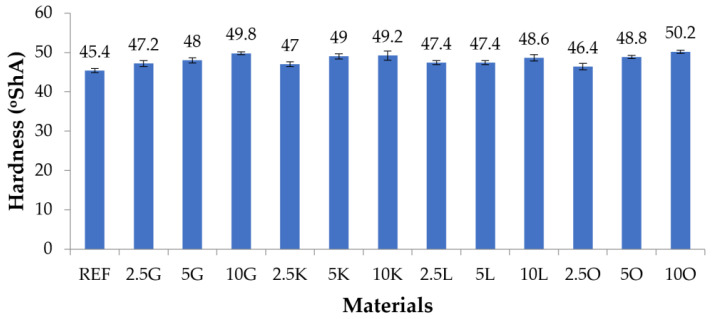
The hardness of the tested materials.

Figure 5 and Table 7. The lowest hardness was measured for the material without added filler (45.4°ShA). The hardness values of composites with citrus fruit fillers were higher than that of the reference material. For composites in which grapefruit, lime, and orange played the filler role, the hardness increased with the filler content in the composite. The 2.5 and 5 wt.% lemon filler composites had the same hardness of 47.4°ShA. The hardness of the 10L composite was 48.6°ShA. The materials with the highest filler content (10 wt.%) in all composites had the highest hardness. The material 10O (50.2°ShA) had the highest hardness, approximately 11% greater than the material without added filler.

### 3.6. Pin-on-Disc

**Figure 6:**
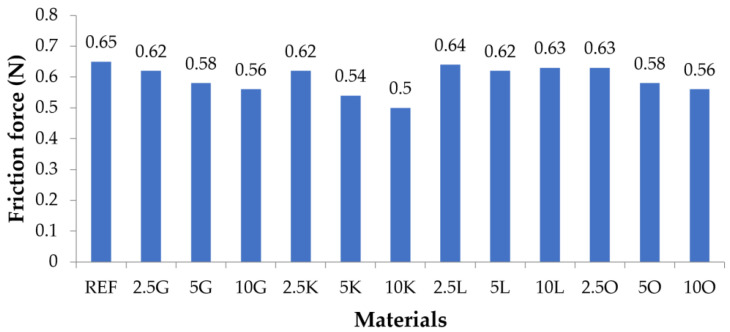
Friction force of the tested materials.

The friction force values are presented in Figure 6 and Table 8. The friction force value of the reference material was 0.65 N, the highest friction force value of all tested materials. Convergence was noticed for composite materials containing grapefruit, lime, and orange in the matrix. For all composite materials, the friction force value decreased upon increasing the filler content in the matrix. The exception to this was composited with lemon filler. The friction force values for the materials were similar, and it should be considered that the friction force values for all lemon-containing materials, regardless of the filler content, were equal (2.5L: 0.64 N, 5L: 0.62 N, and 10L: 0.63 N). The lowest friction force value was recorded for the 10K material (0.5 N), which was 23% lower than the reference sample.

### 3.7. Abrasion Resistance Tests

**Figure 7:**
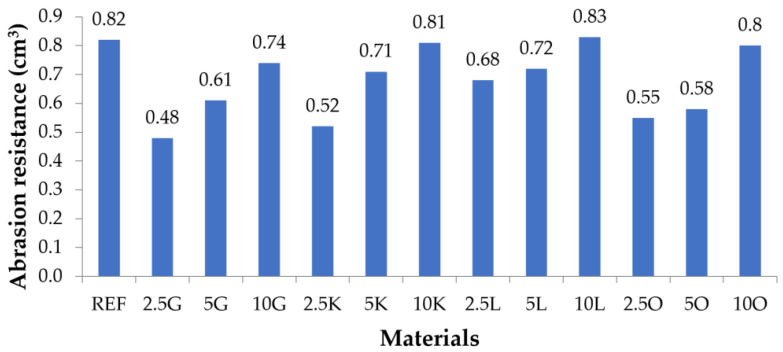
Abrasion resistance of the tested materials.

**Figure A2:**
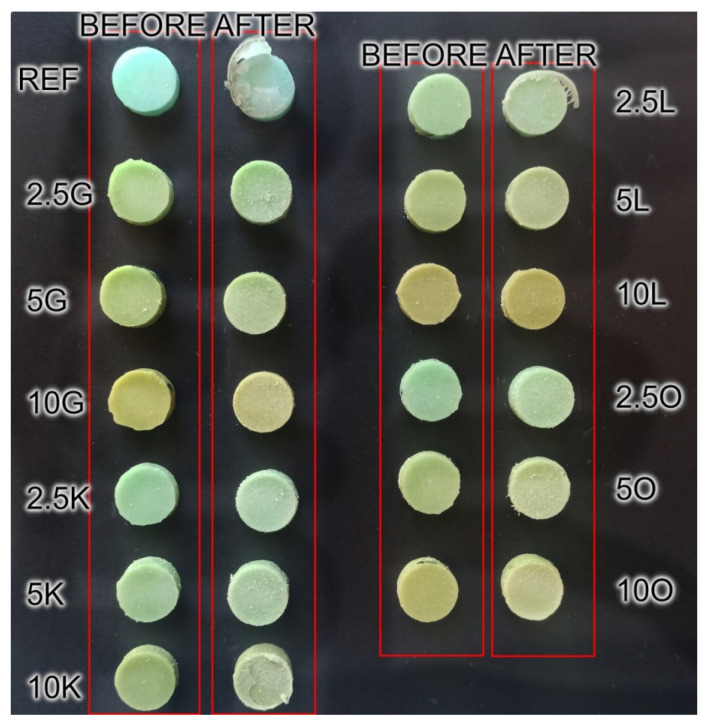
Samples before and after the abrasive wear test.

The results of the abrasion resistance tests are presented in Figure 7 and Table 9. The highest value of abrasive wear was observed for the REF sample (0.82 cm^3^) and for composite materials with 10% filler (10G: 0.74 cm^3^, 10K: 0.81 cm^3^, 10L: 0.83 cm^3^, and 10O: 0.8 cm^3^). These values should be considered equal in the case of REF, 10K, 10L, and 10O. All composite materials’ abrasion resistance decreased upon increasing filler content. The lowest values of abrasive wear were observed for materials with the lowest filler contents (2.5G: 0.48 cm^3^, 2.5K: 0.52 cm^3^, 2.5L: 0.68 cm^3^, and 2.5O: 0.55 cm^3^). The lowest consumption values were recorded for the 2.5G composite. Comparable abrasive wear was observed for 2.5K and 2.5O materials. The lowest abrasive wear value of 0.48 cm^3^ was over 41% lower than the abrasive wear of the REF sample. Figure A2 shows photos of samples before and after the abrasive wear test using the Schopper–Schlobach apparatus.

## 4. Discussion

The article’s authors decided to investigate the effect of food industry waste on the physical properties of silicone-based composites. The choice of silicone as the composite material matrix was dictated by its low price and ease of sample preparation. Ground and dried citrus peels were used as fillers, which belong to the waste that cannot be recycled. The authors used the entirety of the powder obtained during the grinding process of destruction to check the behavior of the composite material into which the filler is completely inserted. This allowed for the management of all waste as it was obtained without separating it into smaller fractions. The obtained composite materials with a filler content of 2.5, 5, and 10 wt.% by mass were tested for the fillers’ influence on the new materials’ physical properties. The results were compared with the properties of silicone without added filler (REF). This research aimed to show a change in the physical properties of the materials, not the commercial application of new materials. More extensive analysis of fillers and composite materials is needed to look deeper into the commercial application of the obtained composite materials.

Two parameters were considered in the conducted mechanical tests: tensile strength and elongation at break. The tensile strength results show that the values of this parameter are the highest for the silicone without adding fillers. The addition of fillers decreased the tensile strength values in each case. For materials with the addition of grapefruit, lemon, and orange, the best deals were recorded for samples with a filler equal to 2.5 wt.% by weight. In the case of materials filled with key lime, the highest value was recorded for the material with 5% filling. For materials with the addition of grapefruit, lemon, and oranges, the tensile strength values decreased along with an increase in the filler content. For materials with the key lime additive, the tensile strength value for the 2.5K material was lower than for the 5K material. Then, however, a decrease in the tensile strength value was noted for the 10K material.

The second parameter examined was elongation at break. For materials in which grapefruit, lemon, and orange acted as the filler, it was observed that the highest elongation at break values were found for composite materials with 2.5 wt.% by mass of the filler, they were lower for materials with 5 wt.% by mass of the filler, and the lowest values for elongation at break were for materials 10 wt.% by a group of the filler. Key lime-filled materials showed a different pattern—a similar elongation at break value characterized materials with 2.5 and 5 wt.% by weight of filler content. Material with 10 wt.% filling in the form of key lime had a higher elongation at break value than materials with 2.5 and 5 wt.% by mass of key lime filling. The 2.5G, 5G, 2.5L, 5L, and 2.5O materials were characterized by a higher elongation at break value than the silicon without adding a filler. In the case of these materials, it can be said that the addition of the filler improved their elongation at break value.

In the case of hardness measurements for materials in which the fillers were grapefruit, key lime, and orange, it was noticed that for all these materials, the hardness value increased with the increase in the amount of filler. For materials in which the filler was lemon, the hardness values for materials with 2.5 and 5 wt.% filling equaled the hardness value. Then, it increased for the material with 10 wt.% filling. All tested composite materials had a hardness more significant than the silicone without adding a filler. In the abrasion resistance studies, it was observed that the highest abrasion resistance was recorded for materials with the lowest percentage of filler. With an increase in the mass content of the filler, the abrasion resistance decreased. For materials 10K, 10L, and 10O, abrasion resistance, comparable to abrasion resistance, was noted for the silicone used as the matrix.

Attention should be paid to the possible influence of the particle size of the fillers on the obtained values of tensile strength and elongation at break. Since the best values for these parameters were obtained for grapefruit and lemon, the yield of grains larger than 0.125 mm was 93.2% and 92.4%, respectively, while for key lime and orange, it was 33.9% and 43.11, respectively. Moreover, in the work of Salih et al. 2019 [49] in which nanopowders of other bio fillers, i.e., ajwa dates seed and pomegranate peels, were used as a filler, the opposite relationships to those presented in this article were obtained. Namely, the addition of the nanopowder increased the tensile strength but worsened the elongation at break. Hence, future work is planned to determine the effect of the level of comminution of selected citrus peels on the mechanical properties of the silicone-based composite. Alsadi et al. examined the influence of the pistachio shell particle on the mechanical properties of composites whose matrix was polyester. The impact content of the pistachio shell particle in the composite was 5, 10, 15, 20, and 25 wt.%. The tests showed that in the case of composites with a content of 5 and 10%, an increase in tensile strength, flexural strength, and impact strength was observed compared to polyester without adding filler. For the filler content of 15, 20, and 25 wt.%, the results of these properties were lower than for neat polyester [50]. Nayak et al. investigated the possibility of using pistachio shell particles with flax fibers as reinforcement in polyester composites. Composites with a 15% mass fraction of fibers with pistachio shell flakes range from 1 to 3 wt.%. The results indicate that adding pistachio shell flakes reduces the tensile properties of the composites while their flexural and impact properties improve [51]. Al-Obaidi et al. investigated the effect of pistachio shell particles with different grain diameters on the mechanical properties of epoxy matrix composites. The research proved that grains of pistachio shell particles of various diameters might improve, deteriorate, or have no effect on tensile, flexural, Izod impact, and hardness tests compared to epoxy resin [52]. Zarrinbakhsh et al. investigated the effect of coffee chaff (CC) and spent coffee grounds (SCG) on the physicochemical properties of polypropylene matrix composites. The conducted tests proved that the introduction of 25 wt.% of the filler content into the composite significantly deteriorated the mechanical properties, such as tensile yield strength, tensile modulus, elongation at yield, and elongation at break compared to polypropylene without the addition of filler [53].

## 5. Conclusions

The conducted research aimed to demonstrate whether adding ground citrus peel could improve composite materials’ physical and mechanical properties with silicone as the matrix. All tested composites had lower tensile strength relative to the silicone matrix. The elongation at break values varied, and for 2.5G, 5G, 2.5L, 5L, 2.5O, and 5O composite materials, they were more significant or comparable to the elongation at break of the REF material. The addition of citrus peel waste increased the hardness of all composites. Composites with 2.5 and 5 wt.% filler displayed increased abrasion resistance relative to the REF sample. Moreover, the addition of fillers did not change the density. This research shows composite materials with 2.5 wt.% grapefruit filler had the best physical properties. This material was characterized by a 41% reduction in abrasion wear compared to the blank test. Moreover, the decrease in the tensile strength was lower in all composites compared with the REF sample (value comparable to 2.5 L). Moreover, for the 2.5G samples, the elongation at break was the highest among all materials tested.

## Figures and Tables

**Table 1:** Composition of samples.

Sample Name	Silicone	Filler	Filler Content (wt.%)
REF	Mold Star 30	-	0
2.5G		grapefruit	2.5
5G		grapefruit	5
10G		grapefruit	10
2.5K		key lime	2.5
5K		key lime	5
10K		key lime	10
2.5L		lemon	2.5
5L		lemon	5
10L		lemon	10
2.5O		orange	2.5
5O		orange	5
10O		orange	10

**Table 2:** Particle size analysis of ground citrus peels.

Grainclass (mm)	Yield of Product (%)			
	Grapefruit	Key Lime	Lemon	Orange
>0.32	4.8	3.81	0.77	0.55
0.32–0.22	2.75	1.52	1.34	0.52
0.22–0.18	45.53	11.7	50.85	4.67
0.18–0.16	19.25	5.64	14.04	10.97
0.16–0.125	20.82	11.21	25.35	26.4
0.125–0.065	5.69	36.16	3.27	32.76
0.065–0.03	0.97	20.98	3.64	18.61
<0.03	0.19	8.98	0.74	5.52

**Table 3:** Bulk and tap densities of the fillers.

Filler	Bulk Density (g/cm^3^)	Tap Density (g/cm^3^)
Grapefruit	0.42	0.69
Key lime	0.48	0.84
Lemon	0.44	0.84
Orange	0.43	0.78

**Table 4:** Tensile strengths of the tested materials.

Materials	REF	2.5G	5G	10G	2.5K	5K	10K	2.5L	5L	10L	2.5O	5O	10O
Tensile strength (MPa)	3.08 ± 0.63	2.84 ± 0.27	2.75 ± 0.46	2.23 ± 0.16	2.48 ± 0.27	2.6 ± 0.41	2.11 ± 0.22	2.85 ± 0.28	2.82 ± 0.44	2.27 ± 0.25	2.63 ± 0.3	2.58 ± 0.46	2.13 ± 0.29

**Table 5:** Elongation at break of the tested materials.

Materials	REF	2.5G	5G	10G	2.5K	5K	10K	2.5L	5L	10L	2.5O	5O	10O
Elongation at break (%)	145 ± 22.45	155 ± 21.76	146 ± 13.37	133 ± 19.06	136 ± 4.04	137 ± 14.99	140 ± 15.87	153 ± 7.77	152 ± 11.75	138 ± 24.98	150 ± 11.9	145 ± 13.89	134 ± 19.06

**Table 6:** The densities of the tested materials are determined by the hydrostatic weighing method.

Materials	REF	2.5G	5G	10G	2.5K	5K	10K	2.5L	5L	10L	2.5O	5O	10O
Density (g/cm^3^)	1.14 ± 0.006	1.15 ± 0.001	1.15 ± 0.012	1.15 ± 0.003	1.14 ± 0.002	1.15 ± 0.004	1.15 ± 0.018	1.14 ± 0.005	1.14 ± 0.003	1.14 ± 0.006	1.14 ± 0.003	1.15 ± 0.002	1.15 ± 0.07

**Table 7:** The hardness of the tested materials.

Materials	REF	2.5G	5G	10G	2.5K	5K	10K	2.5L	5L	10L	2.5O	5O	10O
Hardness (ShA)	45.4 ± 0.49	47.2 ± 0.75	48 ± 0.63	49.8 ± 0.4	47 ± 0.63	49 ± 0.63	49.2 ± 1.17	47.4 ± 0.49	47.4 ± 0.49	48.6 ± 0.8	46.4 ± 0.8	48.8 ± 0.4	50.2 ± 0.4

**Table 8:** Friction force of the tested materials.

Materials	REF	2.5G	5G	10G	2.5K	5K	10K	2.5L	5L	10L	2.5O	5O	10O
Friction force (N)	0.65	0.62	0.58	0.56	0.62	0.54	0.5	0.64	0.62	0.63	0.63	0.58	0.56

**Table 9:** Abrasion resistance of the tested materials.

Materials	REF	2.5G	5G	10G	2.5K	5K	10K	2.5L	5L	10L	2.5O	5O	10O
Abrasion resistance (cm^3^)	0.82	0.48	0.61	0.74	0.52	0.71	0.81	0.68	0.72	0.83	0.55	0.58	0.8

## Data Availability

Not applicable.

## Appendix A

**Table A1:** Properties of Mold Star™ 30 silicone.

Properties	Unit	Value
Ratio mixing silicone: catalyst		1: 1
Pot life	min	45
Cure time	h	6
Density	g/cm^3^	1.12
Viscosity	cps	12,500
Tensile strength	MPa	2.9
Elongation at break	%	150–200
